# Up-regulation of β-amyloidogenesis in neuron-like human cells by both 24- and 27-hydroxycholesterol: protective effect of *N*-acetyl-cysteine

**DOI:** 10.1111/acel.12206

**Published:** 2014-02-25

**Authors:** Paola Gamba, Michela Guglielmotto, Gabriella Testa, Debora Monteleone, Chiara Zerbinati, Simona Gargiulo, Fiorella Biasi, Luigi Iuliano, Giorgio Giaccone, Alessandro Mauro, Giuseppe Poli, Elena Tamagno, Gabriella Leonarduzzi

**Affiliations:** 1 Department of Clinical and Biological Sciences, University of TurinOrbassano, Turin, Italy; 2 Department of Neuroscience “Rita Levi Montalcini”, University of TurinOrbassano, Turin, Italy; 3 Department of Medico-Surgical Sciences and Biotechnology, Vascular Biology and Mass Spectrometry Laboratory, Sapienza University of RomeLatina, Italy; 4 Foundation IRCCS Institute of Neurology Carlo BestaMilan, Italy; 5 Division of Neurology and Neurorehabilitation, IRCCS Italian Institute of AuxologyVerbania, Italy; 6 Department of Neurosciences, University of TurinTurin, Italy

**Keywords:** 27-hydroxycholesterol, 24-hydroxycholesterol, BACE1, amyloid β, Alzheimer’s disease, *N*-acetyl-cysteine

## Abstract

An abnormal accumulation of cholesterol oxidation products in the brain of patients with Alzheimer’s disease (AD) would further link an impaired cholesterol metabolism in the pathogenesis of the disease. The first evidence stemming from the content of oxysterols in autopsy samples from AD and normal brains points to an increase in both 27-hydroxycholesterol (27-OH) and 24-hydroxycholesterol (24-OH) in the frontal cortex of AD brains, with a trend that appears related to the disease severity. The challenge of differentiated SK-N-BE human neuroblastoma cells with patho-physiologically relevant amounts of 27-OH and 24-OH showed that both oxysterols induce a net synthesis of Aβ_1-42_ by up-regulating expression levels of amyloid precursor protein and β-secretase, as well as the β-secretase activity. Interestingly, cell pretreatment with *N*-acetyl-cysteine (NAC) fully prevented the enhancement of β-amyloidogenesis induced by the two oxysterols. The reported findings link an impaired cholesterol oxidative metabolism to an excessive β-amyloidogenesis and point to NAC as an efficient inhibitor of oxysterols-induced Aβ toxic peptide accumulation in the brain.

## Introduction

Life expectancy has increased dramatically in most parts of the world over the last 40 years but, in parallel, the impact of illness and disability on the aging population has also risen significantly. Alzheimer’s disease (AD) is undoubtedly one of the major age-related diseases, and its incidence continues increasing at an alarming rate.

The underlying multifactorial and multistep disease process, characterized by brain accumulation of extracellular amyloid-β (Aβ) peptide plaques and intracellular neurofibrillary tangles, is often preceded and/or accompanied by other important morbidities, including abdominal obesity, insulin resistance, and altered cholesterol metabolism (Gamba *et al*., 2012; Reitz, 2012).

While the contribution made by altered brain cholesterol metabolism to the complex pathogenesis of AD has recently gained further consensus, the mechanisms linking this metabolic impairment to the hallmark lesions of AD, that is, extracellular Aβ deposits and intraneuronal tau pathology, have not yet been clarified.

To date, most research on this point has focused on the ability of cholesterol to modulate amyloidogenesis, that is, Aβ production, in the brain. In this connection, experimental studies carried out thus far, using cell culture systems and/or animal models, have consistently proved that excess cholesterol may stimulate amyloidogenesis by neuronal cells and that hypercholesterolemia is associated with increased deposition of Aβ in the brain (for a review, see Ricciarelli *et al*., 2012). In one such study, a long-term dietary regimen rich in cholesterol not only augmented plasma cholesterol in rabbits but also increased the cholesterol content in the animal’s neurons. In parallel, the level of neuronal β-secretase, the enzyme cleaving amyloid precursor protein (APP) so as to generate Aβ, was found to be increased, as was the level of Aβ itself (Ghribi *et al*., 2006). Rats fed a cholesterol-rich diet for 5 months showed impaired spatial memory, together with a significant loss of cholinergic neurons. These findings were associated with increased levels of APP, Aβ, and phosphorylated tau in the cerebral cortex. Importantly, this dietary regimen was demonstrated to derange the semi-permeability of the blood–brain barrier (Ehrlich & Humpel, 2012).

Thus, at least in certain experimental animals, hypercholesterolemia may somehow favor an actual increase in neuron cholesterol content, one operated mechanism being modulation of the cellular processing of APP (Ghribi, 2008; Schweinzer *et al*., 2011). However, epidemiological studies relating high plasma cholesterol levels to AD, and clinical trials with hypocholesterolemic drugs, have thus far given controversial results (Reitz, 2012; Ricciarelli *et al*., 2012).

Of note, whereas abnormalities in cholesterol metabolism are tied to a derangement of cholesterol synthesis and uptake in the peripheral tissues, leading to increased ‘total’ plasma cholesterol, that is, hypercholesterolemia, in many cases, they also appear to involve oxidative modification of cholesterol and/or altered cholesterol homeostasis within the brain. As we know, this compound is essential for brain structure and function and the cholesterol content of the brain accounts for about the 25% of the total body content (Björkhem & Meaney, 2004). In our view, the AD-predisposing role played by homozygosity for the apolipoprotein E (APOE) e4 allele (Evans *et al*., 2004) is likely just one of several ways in which abnormal brain cholesterol metabolism may contribute to the development of this disease.

A key role in the regulation of cholesterol homeostasis in the brain is undoubtedly played by the biochemical events that regulate its oxidation rate. In general, the production of cholesterol oxidation products in the body, particularly that of oxysterols, may be either enzymatic or nonenzymatic (Leonarduzzi *et al*., 2002; Brown & Jessup, 2009; Sottero *et al*., 2009; Iuliano, 2011). In the brain, the enzymatic source of oxysterols greatly prevails, at least under physiological conditions; through this process, the brain can release excess cerebral cholesterol into the blood stream. Whereas the normal blood–brain barrier is not permeable to cholesterol as such, it thus allows the diffusion of at least some cholesterol oxidation products, for example 24-hydroxycholesterol (24-OH) and 27-hydroxycholesterol (27-OH) (Björkhem *et al*., 2009). Mainly for this reason, scientists initially tended to consider oxidation of brain cholesterol as a beneficial event. However, it cannot be ruled out that, under pathological conditions like those leading to AD, steady-state levels of oxysterols in the brain may overwhelm the brain’s capacity to expel these compounds. In this connection, most oxysterols have shown 10–100 times stronger biochemical reactivity than the parent compound, often exhibiting quite strong pro-apoptotic and pro-inflammatory effects (Poli *et al*., 2009; Vejux & Lizard, 2009).

Significantly increased levels of 24-OH have been found in the cerebrospinal fluid of patients with AD (Schönknecht *et al*., 2002); levels of another oxysterol of enzymatic origin, 27-OH, were increased in the frontal cortex of patients with AD versus control individuals, while the amount of frontal cortex 24-OH recovered in the same patients did not show any significant difference as to over controls (Heverin *et al*., 2004). The two enzymes catalyzing cholesterol oxidation into 24-OH or into 27-OH, respectively, 24-cholesterol hydroxylase (CYP46) and 27-cholesterol hydroxylase (CYP27), showed an abnormal pattern in the AD brain, with increased expression of 24-cholesterol hydroxylase in the neighborhood of amyloid plaques (Brown *et al*., 2004).

In the light of these findings, 24-OH and 27-OH have been the two main oxysterols considered over the last few years for their potential neurodegenerative action. However, to date, few *in vitro* studies have focused on the possible implication of these two cholesterol oxidation products in amyloidogenesis. A significant up-regulation of the APP level (3.2-fold induction vs. control cells) was observed in primary cultures of almost equal populations of human neuronal and glial cells, after incubation in the presence of 24-OH (10 μm final concentration) (Alexandrov *et al*., 2005). Following the treatment of the undifferentiated human neuroblastoma cell line SH-SY5Y with either 24-OH or 27-OH, both employed at 5 μm final concentration, a significant doubling of α-secretase and down-regulation of β-secretase (BACE1) activities occurred in the presence of 24-OH, whereas 27-OH-treated cells behaved like controls (Famer *et al*., 2007). Another research group, adopting the same cell model system (SH-SY5Y), showed that 27-OH (5–15 μm final concentration) was significantly able to up-regulate cell APP levels and BACE1 activity, while identical concentrations of 24-OH did not show any significant effect either on APP levels or on BACE1 activity. The effect of the two oxysterols on α-secretase was not reported, but the possibility that 24-OH stimulated the nonamyloidogenic pathway was supported by a net increase in sAPPα secretion by SH-SY5Y treated with the latter compound (Prasanthi *et al*., 2009).

The present study comprises a comprehensive *in vitro* analysis of APP, α- β- and γ-secretase expression and levels, and β- and γ-secretase activities, all measured in a human neuroblastoma cell line (SK-N-BE); most importantly, the cells were first differentiated toward a neuronal phenotype, by treatment with all-*trans*-retinoic acid, then challenged with ‘patho-physiological amounts’ of 24-OH or 27-OH. The latter experimental condition was determined on the basis of the quantification of these two oxysterols in a few postmortem samples of brains with different levels of Alzheimer pathology within a pilot analysis that might suggest their increasing trend in the AD brain frontal cortex with disease progression. Aβ production by differentiated SK-N-BE cells, under treatment with 24-OH or 27-OH, was also investigated, as well as its potential modulation by cell pretreatment with *N*-acetyl-cysteine (NAC), a redox active molecule of clinical interest.

## Results

### Levels of 27-OH and 24-OH in the frontal cortex of AD brains: upward trend with disease progression

A pilot study was carried out on autopsy samples of frontal cortex from AD brains partly to obtain reliable indications concerning the appropriate concentration of 27-OH and 24-OH to use in the *in vitro* experiments scheduled subsequently. As reported in Table 1, in control brain samples, the average amounts of 27-OH and 24-OH recovered were about 0.2 and 2.5 ng mg^−1^ of tissue, respectively. Interestingly, when a distinction was made between early and advanced AD cases, following the classification of Braak and Braak (see Experimental procedures), the steady-state amounts of the two oxysterols recovered from the cerebral frontal cortex might increase with disease progression. When AD data were grouped together, not considering the disease stage of the donors, and compared to controls, frontal cortex 27-OH and 24-OH levels were, respectively, triple and double those of normal frontal cortex samples (Table 1).

**Table 1:** Quantification of 27-hydroxycholesterol (27-OH) and 24-hydroxycholesterol (24-OH) in autopsy samples of frontal cortex from AD brains

	27-OH (ng mg^−1^)	24-OH (ng mg^−1^)
Control	0.2 ± 0.02	2.5 ± 0.14
Early AD	0.4 ± 0.10	3.3 ± 0.04
Late AD	0.9 ± 0.32**,#	7.6 ± 2.86*,#
Total AD	0.7 ± 0.39*	5.6 ± 2.8*

AD, Alzheimer’s disease.

Early AD (Braak and Braak stages 1, 2); late AD (Braak and Braak stages 4, 6). Control brain samples: *n* = 4; early AD samples: *n* = 6; and late AD samples: *n* = 6.

*P < 0.05, and **P < 0.01 versus control; ^#^*P* < 0.05 versus early AD.

Based on the amounts of 27-OH and 24-OH actually detected in AD and normal autopsy brains, given the molecular weight of 27-OH and 24-OH (M.W. 402.7 g mol^−1^), the final concentration of 1 μm was deemed the most logical one to adopt for the *in vitro* analysis of amyloidogenesis in neuroblastoma-derived cells under challenge with oxysterols.

### 27-OH and 24-OH up-regulate APP level in differentiated SK-N-BE human neuroblastoma cells

**Figure 1:**
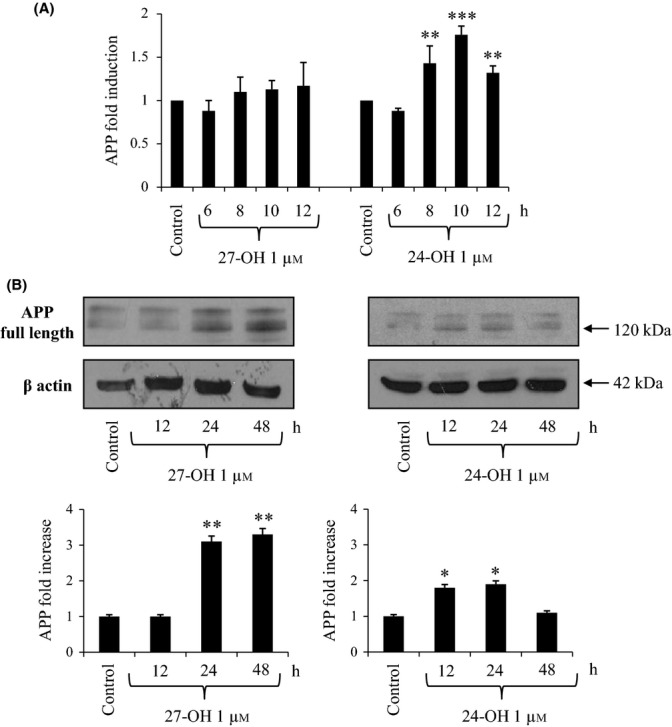
Effect of 27-hydroxycholesterol (27-OH) and 24-hydroxycholesterol (24-OH) on the expression and synthesis of the amyloid precursor protein (APP). (A) Gene expression was quantified by real-time RT–PCR in differentiated SK-N-BE cells treated for times up to 12 h with 1 μm 27-OH or 24-OH. Untreated cells were taken as control. Data, normalized to β2-microglobulin, are expressed as mean values ± SD of four different experiments. **P < 0.01, and ***P < 0.001 versus control group. (B) APP protein levels were analyzed by Western blotting in differentiated SK-N-BE cells treated up to 48 h with 1 μm 27-OH or 24-OH. Untreated cells were taken as control. APP densitometric measurements were normalized against the corresponding β actin levels. The experiments were conducted in triplicate. *P < 0.05, and **P < 0.01 versus control group.

The initial experiments, upon SK-N-BE differentiated into more neuron-like cells by treatment with all-*trans*-retinoic acid (see Experimental procedures), then incubated in the presence or absence of 27-OH or 24-OH at the selected final concentration of 1 μm, verified the expression and level of APP. Only 24-OH-treated cells showed statistically significant overexpression of the amyloid precursor (Fig. 1A). Both oxysterols were in any case able to significantly increase the steady-state cellular concentration of APP protein (Fig. 1B).

### 27-OH and 24-OH up-regulate BACE1 level in differentiated SK-N-BE cells

**Figure 2:**
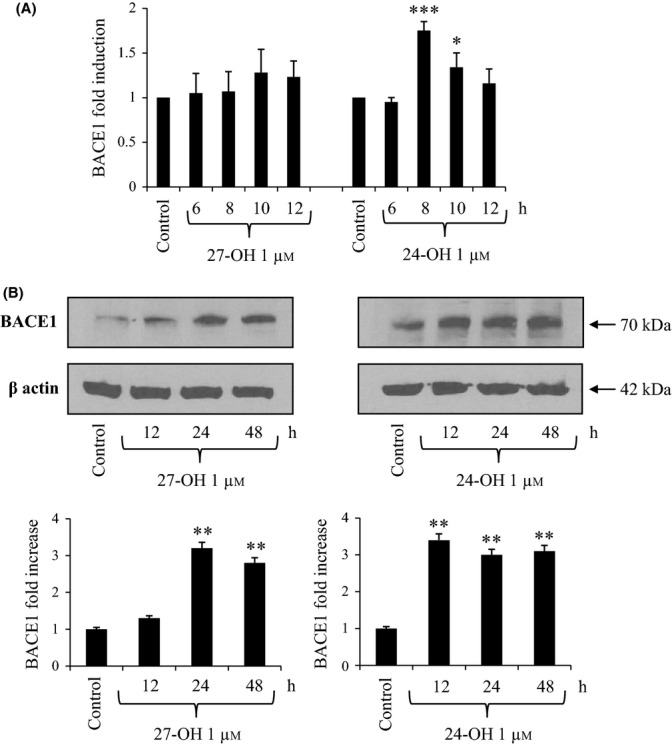
Effect of 27-hydroxycholesterol (27-OH) and 24-hydroxycholesterol (24-OH) on the expression and synthesis of β-secretase (BACE1). (A) Gene expression was quantified by real-time RT–PCR in differentiated SK-N-BE cells treated for times up to 12 h with 1 μm 27-OH or 24-OH. Untreated cells were taken as control. Data, normalized to β2-microglobulin, are expressed as mean values ± SD of four different experiments. *P < 0.05, and ***P < 0.001 versus control group. (B) BACE1 protein levels were analyzed by Western blotting in SK-N-BE cells treated up to 48 h with 1 μm 27-OH or 24-OH. Untreated cells were taken as control. BACE1 densitometric measurements were normalized against the corresponding β actin levels. The experiments were conducted in triplicate. **P < 0.01 versus control group.

As shown in Fig. 2A, 27-OH (1 μm final concentration) did not appear to significantly increase BACE1 mRNA levels, while treatment with the same concentration of 24-OH induced a 1.5-fold to twofold increase, which became statistically significant after 8- to 10-h cell incubation. However, both oxysterols up-regulated the secretase protein level. In fact, SK-N-BE treatment with 27-OH was followed by a statistically significant increase in BACE1 protein levels (almost tripling them) after 24- and 48-h cell incubation. In line with the mRNA results, 24-OH-challenged cells showed an earlier increase (3.5-fold) in BACE1 protein levels, which was already significant after 12-h incubation (Fig. 2B).

### 27-OH, but not 24-OH, increases expression and synthesis of γ-secretase catalytic unit presenilin-1

**Figure 3:**
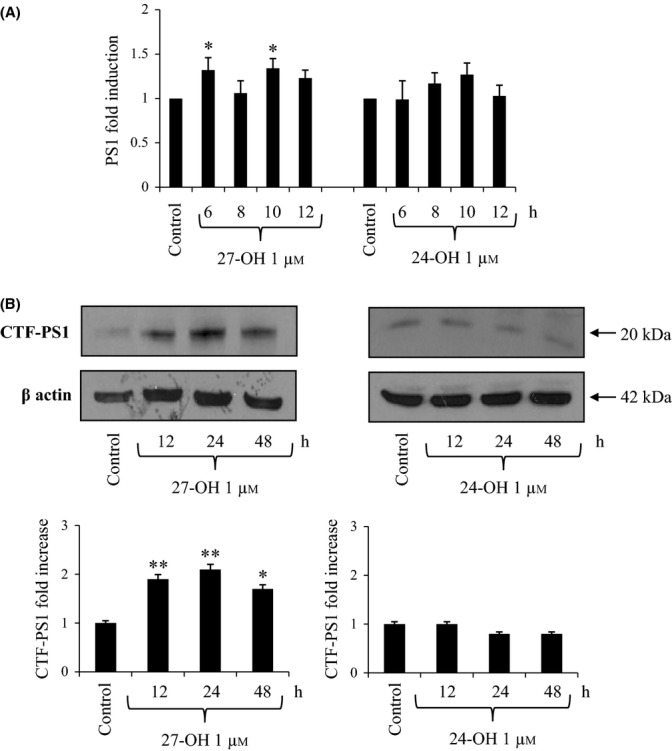
Effect of 27-hydroxycholesterol (27-OH) and 24-hydroxycholesterol (24-OH) on the expression and synthesis of the γ-secretase subunity presenilin 1 (PS1). (A) Gene expression was quantified by real-time RT–PCR in SK-N-BE cells treated for times up to 12 h with 1 μm 27-OH or 24-OH. Untreated cells were taken as control. Data, normalized to β2-microglobulin, are expressed as mean values ± SD of four different experiments. *P < 0.05 versus control group. (B) The C-terminal fragment (CTF) of PS1 (CTF-PS1) levels were analyzed by Western blotting in SK-N-BE cells treated up to 48 h with 1 μm 27-OH or 24-OH. Untreated cells were taken as control. CTF-PS1 densitometric measurements were normalized against the corresponding β actin levels. The experiments were conducted in triplicate. *P < 0.05, and **P < 0.01 versus control group.

To test the effect of the two oxysterols on γ-secretase, expression and protein levels of presenilin-1 (PS1), that is, the catalytic unit of γ-secretase, were determined. Real-time RT–PCR revealed that, in differentiated SK-N-BE neuroblastoma cells, a single treatment with 27-OH (1 μm) induced a statistically significant increase (1.5-fold) in PS1 mRNA levels compared to untreated cells; conversely, cell treatment with 24-OH (1 μm) did not modify basal PS1 mRNA levels (Fig. 3A). PS1 protein level results were fully consistent with those obtained by real-time RT–PCR: 27-OH significantly increased the C-terminal fragment (CTF) of PS1 (CTF-PS1) levels (doubling them) in SK-N-BE cells, from 12- up to 48-h treatment, while 24-OH did not show any effect (Fig. 3B).

### 27-OH and 24-OH up-regulate expression and synthesis of α-secretase

**Figure 4:**
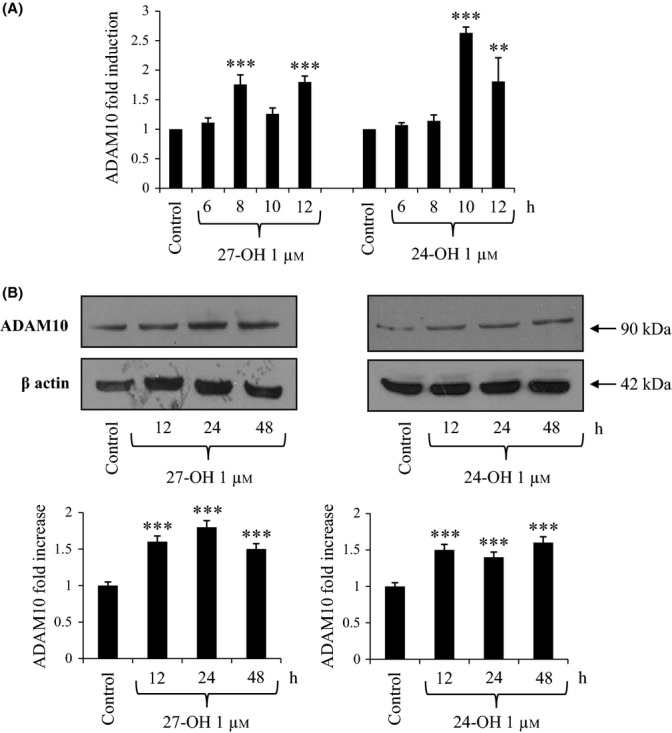
Effect of 27-hydroxycholesterol (27-OH) and 24-hydroxycholesterol (24-OH) on the expression and synthesis of α-secretase (ADAM10). (A) Gene expression was quantified by real-time RT–PCR in differentiated SK-N-BE cells treated for times up to 12 h with 1 μm 27-OH or 24-OH. Untreated cells were taken as control. Data, normalized to β2-microglobulin, are expressed as mean values ± SD of four different experiments. **P < 0.01, and ***P < 0.001 versus control group. (B) ADAM10 protein levels were analyzed by Western blotting in SK-N-BE cells treated up to 48 h with 1 μm 27-OH or 24-OH. Untreated cells were taken as control. ADAM10 densitometric measurements were normalized against the corresponding β actin levels. The experiments were conducted in triplicate. ***P < 0.001 versus control group.

To evaluate the ability of 27-OH and 24-OH to modulate α-secretase, we measured expression and protein levels of the main enzyme with α-secretase activity in neurons, that is, ADAM10 (a disintegrin and metalloproteinase domain-containing protein 10). ADAM10 mRNA levels in differentiated SK-N-BE cells were found to be significantly increased by 1 μm 27-OH and 24-OH, compared to untreated cells, with a maximum of twofold and 2.5-fold induction, respectively (Fig. 4A). In addition, ADAM10 synthesis was markedly up-regulated (+50%) by both oxysterols from 12- up to 48-h treatment (Fig. 4B).

### Both 27-OH and 24-OH up-regulate BACE1 enzymatic activity; 27-OH also stimulates γ-secretase enzymatic activity

**Figure 5:**
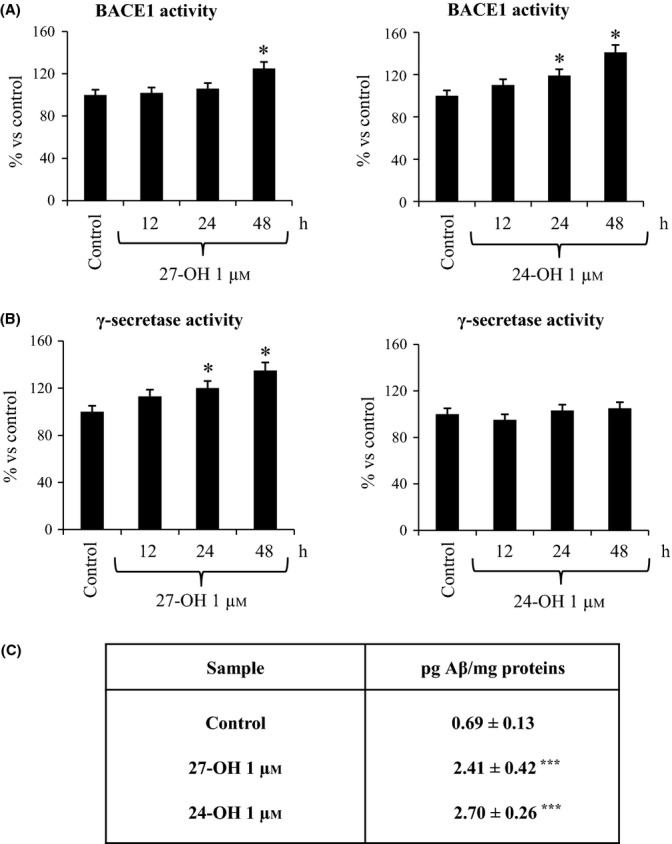
27-hydroxycholesterol (27-OH) and 24-hydroxycholesterol (24-OH) induce Aβ_1-42_ production by up-regulating BACE1 and γ-secretase enzymatic activities in SK-N-BE cells. Differentiated SK-N-BE cells were incubated up to 48 h with 27-OH or 24-OH. Untreated cells were used as control. BACE1 activity (A) and γ-secretase activity (B) were measured by fluorogenic assay using the secretase-specific substrate conjugated to the fluorescent reporter molecules. Data were expressed as percentage change versus activity of control cells. Data are means ± SD of three experiments. *P < 0.05 versus control group. (C) Differentiated SK-N-BE cells were incubated for 24 h with 27-OH or 24-OH. Untreated cells were used as control. Aβ_1-42_ intracellular concentration was quantified by enzyme-linked immunoassay (ELISA). Data are means ± SD of three experiments. ***P < 0.001 versus control group.

In a subsequent step, BACE1 and γ-secretase activities were quantified in differentiated SK-N-BE neuroblastoma cells challenged with a single dose of either 27-OH or 24-OH (1 μm). As shown in Fig. 5A, BACE1 activity was found to be significantly increased (+25%) in 27-OH-treated cells, but only after 48-h treatment; a statistically significant increase of BACE1 activity was evident after 24-h (+20%) and 48-h (+40%) incubation with 24-OH.

The results on γ-secretase activity paralleled those obtained by PS1 expression: γ-secretase activity was significantly increased in differentiated SK-N-BE cells after treatment with 27-OH (+20% after 24 h; +35% after 48 h). As expected, 24-OH did not modify γ-secretase activity (Fig. 5B).

### 27-OH and 24-OH markedly stimulate Aβ_1-42_ production by differentiated SK-N-BE neuroblastoma cells

To fully validate the observed stimulating effect of both 27-OH and 24-OH on APP processing, an ELISA kit procedure was used to quantify the intracellular concentration of Aβ_1-42_, the most toxic and fibrillogenic form of Aβ, before and after oxysterol challenge. Data reported in Fig. 5C, clearly indicate that both oxysterols were able to induce a net increase in Aβ_1-42_ production by SK-N-BE cells; production was found to be about 3–4 times higher than in untreated cells.

In an additional set of experiments, the effect of the oxysterol concentration used in this study (1 μm) was compared to the previously published ones (5 and 10 μm) with regard to Aβ_1–42_ production, the most critical point of the overall work, in both differentiated and undifferentiated SK-N-BE cells (see Fig. S1). In differentiated cells, the ELISA quantification of Aβ_1-42_ confirmed that the treatment with 1 μm 27-OH or 24-OH induced about a fourfold increase in the toxic peptide production, while higher concentrations of the oxysterols (5 and 10 μm) did not show any statistically significant effect. In undifferentiated cells, only the treatment with 5 μm 27-OH showed a statistically significant but moderate increase (+50%) in Aβ_1-42_; conversely, 1 μm 27-OH, 1 and 5 μm 24-OH did not affect the Aβ constitutive amount which is relatively lower than that found in differentiated control cells. At the higher oxysterol concentration tested (10 μm), the amounts of Aβ_1-42_ detected within undifferentiated cells showed lower but not statistically significant values compared to controls (Fig. S1).

### NAC prevents the up-regulation of β- and γ-secretases, as well as the over-production of Aβ_1-42_, in SK-N-BE cells challenged with either 27-OH or 24-OH

Differentiated SK-N-BE cells were incubated in the presence of the strong redox active and antioxidant compound NAC, to investigate whether a redox imbalance was also implicated in the observed pro-amyloidogenic effect exercised by 27-OH and 24-OH.

**Figure 6:**
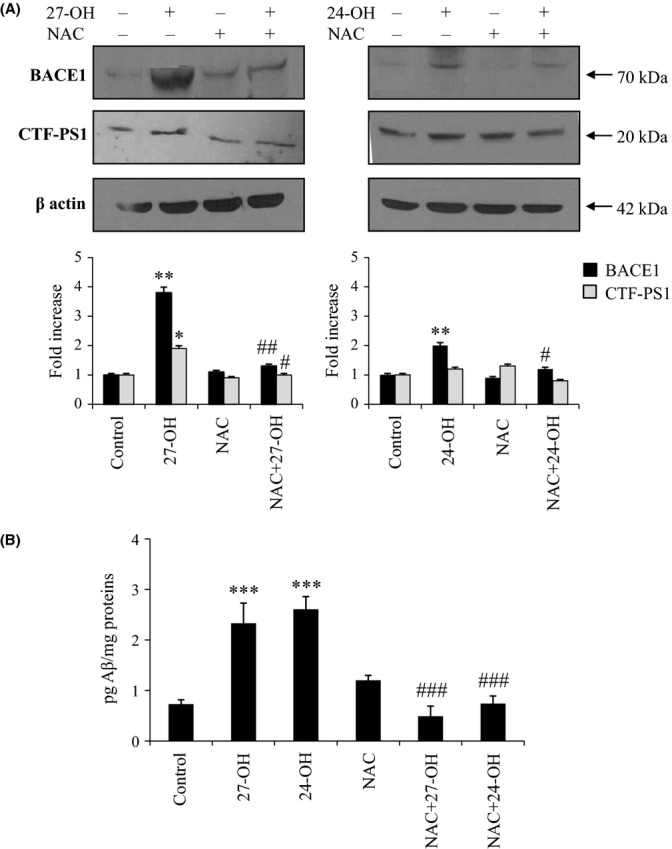
Up-regulation of BACE1 and γ-secretase and Aβ_1-42_ over-production are prevented by cell pretreatment with *N*-acetyl-cysteine (NAC). Differentiated SK-N-BE cells were incubated for 24 h with 27-hydroxycholesterol (27-OH) or 24-hydroxycholesterol (24-OH). Some cell aliquots were also pre-incubated for 1 h with 100 μm NAC. Untreated cells were used as control. (A) The C-terminal fragment (CTF) of PS1 (CTF-PS1) and BACE1 protein levels were analyzed by Western blotting. CTF-PS1 and BACE1 densitometric measurements were normalized against the corresponding β actin levels. The experiments were conducted in triplicate. *P < 0.05, and **P < 0.01 versus control group; #*P* < 0.05, and ##*P* < 0.01 versus oxysterol groups. (B) Aβ_1-42_ intracellular concentration was quantified by enzyme-linked immunoassay (ELISA). Histograms represent the mean values ± SD of three experiments. ***P < 0.001 versus control group, and ^###^*P* < 0.001 versus 27-OH or 24-OH.

The protective action exerted by NAC was demonstrated to be essentially dependent on this thiol compound’s complete prevention of 27-OH- and 24-OH-induced up-regulation of BACE1 protein levels (Fig. 6A). Consistent with these latter findings was the prevention of 27-OH-induced increase in PS1 intracellular levels observed in differentiated SK-N-BE cells pretreated with NAC (Fig. 6A).

In this series of experiments, again, challenge of the neuron-like SK-N-BE cells with either 27-OH or 24-OH induced a marked increase in the steady-state concentration of intracellular Aβ_1-42_. However, the most interesting finding was that the β-amyloidogenic effect exerted by the two oxysterols on differentiated SK-N-BE cells was completely prevented when cell aliquots were incubated for 1 h in the presence of 100 μm NAC, prior to challenge with the cholesterol oxides (Fig. 6B).

## Discussion

Despite general agreement concerning the significant contribution made by deranged brain cholesterol metabolism to the onset and progression of AD, both in the familial form and also in the commoner sporadic form, this metabolic impairment has not recently been investigated in depth. Systematic studies of this and other structural and metabolic changes in the brain of patients with AD, as well as conclusive diagnoses, are today only available postmortem after autoptic inspection; furthermore, proper identification and quantification of cholesterol metabolites in human tissues require sophisticated instruments [gas chromatography–mass spectrometry (GC–MS)] and relatively complex methods of tissue preparation and analysis.

As the oxidation rate of cholesterol is without doubt crucial for the sterol’s homeostasis in the brain, and as excess amounts of cholesterol oxidation products, particularly of oxysterols, have been shown to be detrimental to various types of cells and tissues (Poli *et al*., 2013), it would be of primary interest to know whether specific oxysterols do accumulate in AD brains, and if possible, to discriminate such findings between early and advanced disease stages.

The data reported here are from a pilot study on a limited number of autopsy samples, of brains in which the presence of AD neuropathology has been confirmed by immunohistochemical methods. A net accumulation of both 27-OH and 24-OH was detected in the frontal cortex of all AD brains examined, compared to autopsy samples of frontal cortex from control brains (Table 1). The frontal cortex, as other neocortical regions, is early involved by Aβ deposits in AD, while the hippocampus is site of early neurodegeneration and formation of neurofibrillary changes, but exhibits consistent Aβ lesions only at later stages (Thal *et al*., 2002). We then chose to examine the frontal cortex, because the study’s main aim was to investigate the relationship between Aβ and cholesterol metabolism.

Of interest, in the brains that we used as controls, we excluded the presence of Aβ deposition, ruling out the possibility that they represent nondemented elderly subjects with significant number of Aβ deposits. Even more interestingly, there was an upward trend of 27-OH and 24-OH accumulation with progression of the level of Braak and Braak staging of neurofibrillary pathology (Table 1). Although the small number of samples analyzed thus far does not allow any definitive conclusions to be drawn, the results of this pilot study appear of sufficient significance to support the implication of an altered cholesterol oxidative metabolism in the pathogenesis of sporadic AD.

To our knowledge, only one study has addressed the quantitative measurement of 27-OH and 24-OH levels in the brain cortex of patients with AD. That study showed a net increase only of 27-OH in the frontal cortex of AD brains compared to age-matched normal ones, while 24-OH levels in AD frontal cortex specimens were reported to be unchanged (Heverin *et al*., 2004). Those data were obtained from a similar number of cases, namely eight AD autopsy samples, and by applying virtually the same assay procedure, that is, isotope dilution mass spectrometry. However, the values were one order of magnitude higher than those found in the present study. Levels of 27-OH and 24-OH in the frontal cortex from normal brains were reported to be in the range of 1–2 and 18–20 ng mg^−1^ tissue, respectively (Heverin *et al*., 2004), while in our study, the corresponding average values were 0.1–0.2 ng mg^−1^ tissue 27-OH and 2 ng mg^−1^ tissue 24-OH (Table 1).

Besides providing very useful suggestions for *in vitro* tests of patho-physiologically relevant amounts of brain oxysterols, the oxysterol quantification in brain frontal cortex reported here points to an increase in 27-OH and 24-OH in the cortex of AD brain versus normal brains, with a trend that appears related to the disease severity.

With regard to the *in vitro* investigation of the potential pro-β-amyloidogenic effect of 27-OH and 24-OH, the present study differs from previous analogous ones essentially in two ways: the cell line employed, and the chosen final concentration of the two oxysterols. Other studies into the effect of one or both oxysterols on APP processing used the human neuroblastoma-derived cell line SH-SY5Y, except for one study employing human neural cells (HN cells) in primary culture (Alexandrov *et al*., 2005). The latter report was the only one to show a marked induction of APP protein by cell challenge with 10 μm 24-OH; the few other data available on the effect of 24-OH on APP protein levels (Prasanthi *et al*., 2009) and β-amyloidogenesis (Famer *et al*., 2007; Prasanthi *et al*., 2009) either found no effect or even found a protective effect of this oxysterol. Concerning 27-OH, it has been shown that this oxysterol, at the final concentration of 10 μm, significantly reduced Aβ peptide production in primary human neurons (Kim *et al*., 2009), while in other papers, in 27-OH-treated SH-SY5Y cells, APP processing was found either similar to control values (Famer *et al*., 2007) or significantly enhanced (Prasanthi *et al*., 2009). SH-SY5Y cells were in any case directly challenged with the investigated oxysterols, without prior retinoic acid-driven differentiation toward a more neuron-like phenotype. Conversely, in SK-N-BE cells, ten days of 10 μm all-*trans*-retinoic acid exposure induced evident markers of neuronal differentiation, both morphological and biochemical (Melino *et al*., 1997). In particular, already within 7 days of cell medium supplementation with all-*trans*-retinoic acid, neuroblastoma-derived cells show a neuron-like phenotype (Chambaut-Guérin *et al*., 1995), as confirmed by increased expression levels of the specific differentiation markers GAP-43 (Silvagno *et al*., 2002), NF-200, and NeuN (Redova *et al*., 2010). The other peculiarity of the present study is the lower oxysterol final concentration adopted (1 μm) then that used in other studies, which were in the 5–10 μm range. On the basis of the actual amounts of 27-OH and 24-OH recovered from normal and AD brains, it may be concluded that the 1 μm concentration of these oxysterols is much closer to the actual patho-physiological amount.

Both 27-OH and 24-OH (1 μm) were demonstrated to induce accelerated APP processing toward β-amyloidogenesis in differentiated SK-N-BE cells: both oxysterols significantly up-regulated APP intracellular levels (Fig. 1), and, more importantly, stimulated BACE1 protein levels (Fig. 2), the crucial enzyme in Aβ production. Interestingly, while 24-OH was shown to stimulate both expression and synthesis of APP and BACE1, the effect of 27-OH on the cellular levels of the two proteins appeared to be essentially post-translational.

These findings were corroborated by the up-regulation of BACE1 enzymatic activity (Fig. 5A), and the markedly increased levels of the Aβ_1-42_ peptide that were consistently detectable within differentiated SK-N-BE cells, challenged with either 27-OH or 24-OH (Fig. 5C). Thus, both oxysterols definitely stimulated β-amyloidogenesis at least in the experimental system employed, despite the fact they showed a parallel ability to up-regulate expression and synthesis of ADAM10 (α-secretase), although it is known to be a protective enzyme (Fig. 4).

In all previous investigations on the pro-amyloidogenic effect of 27-OH and/or 24-OH, only undifferentiated neuroblastoma cell lines and relatively higher oxysterol concentrations (5–10 μm) were used. Here, reported comparative measurements of Aβ_1-42_ synthesis in differentiated and undifferentiated SK-N-BE cells clearly point to 1 μm oxysterol amount and differentiated cells as the most efficient concentration and the most convenient cell type to adopt for this kind of study. Challenge of differentiated cells with either 1 μm 27-OH or 1 μm 24-OH was, in fact, the only experimental condition consistently showing a very strong enhancement of toxic Aβ production (Fig. S1). By the way, the findings reported in Fig. S1 (Supporting information) were in agreement with those obtained by Prasanthi *et al*. (2009) who showed that 5–10–25 μm 27-OH, but not 24-OH, stimulated the synthesis of the toxic Aβ peptide in undifferentiated human neuroblastoma cells (SH-SY5Y). Very recently, a markedly decreased synthesis of Aβ_1-40_ and a moderate reduction in the synthesis of Aβ_1-42_ were observed in undifferentiated SH-SY5Y incubated 24 h in the presence of 24-OH (1–10 μm) (Urano *et al*., 2013). All other reports only focused on specific aspects of the modulation of the amyloidogenic pathway by 27-OH and/or 24-OH without quantifying the levels of the toxic peptide.

Indeed, 1 μm 27-OH/24-OH appears to be the closest concentration to that found in human AD brain (see above, Results section); moreover, using differentiated neuroblastoma cell lines is a more convenient experimental model than employing undifferentiated cells of ‘neural’ origin, as cell differentiation with all*-trans-*retinoic acid allows the re-expression of many morphologic and biochemical features that make cells quite similar to normal ‘neuronal’ cells (Chambaut-Guérin *et al*., 1995; Melino *et al*., 1997; Silvagno *et al*., 2002; Redova *et al*., 2010).

Even if the conclusions drawn from *in vitro* studies cannot be directly applicable to neuronal cells *in vivo*, the results obtained appear to be of sufficient significance to suggest their possible *in vivo* relevance. Under specific conditions and concentrations in the brain, not only 27-OH but also 24-OH might exert detrimental effects on neural and neuronal cells. In this connection, at least 24-OH was recently shown to potentiate Aβ_1-42_-induced apoptotic and necrotic death in differentiated SK-N-BE and NT-2 neuron-like cells (Gamba *et al*., 2011) as well as in human dental pulp-derived cells showing a neuron-like phenotype (Testa *et al*., 2012).

Finally, with regard to the observed complete inhibition of 27-OH- and 24-OH-dependent stimulation of BACE1 level and Aβ production in SK-N-BE cells pretreated with NAC (Fig. 6), a possible involvement of oxysterol-mediated redox impairment is hypothesized. On the one hand, both expression and levels of BACE1 have been shown to be up-regulated by oxidative stress conditions and lipid peroxidation end products (Tamagno *et al*., 2003; Huang *et al*., 2013), and the pro-amyloidogenic processing has been found to be inhibited by a number of polyphenolic compounds, all provided with strong antioxidant effects (Shimmyo *et al*., 2008; Williams & Spencer, 2012). Moreover, a growing bulk of experimental evidence points to oxysterols as potential inducers of reactive oxygen species (ROS), either by inducing different isoforms of the NADPH oxidase, or by deranging the mitochondrial membrane potential (Pedruzzi *et al*., 2004; Biasi *et al*., 2009; Gamba *et al*., 2011; Biasi *et al*., 2013).

In conclusion, we have found a low micromolar amount of 24-OH and 27-OH, the two main oxysterols with potential neurodegenerative action, in the frontal cortex of postmortem samples from normal brains. This concentration was consistently increased in AD brain. Even if the mechanism/s underlying the observed accumulation of these oxysterols in AD brain are not yet completely understood, a redox state impairment in the brain developing the disease seems to play a central role. Oxidative stress is, indeed, recognized as being involved in the onset of AD (Sayre *et al*., 2001; Texel & Mattson, 2011; Rodrigues *et al*., 2012); in this connection, we found that neuronal cell pretreatment with the redox active molecule NAC prevents 27-OH- and 24-OH-induced β-amyloidogenesis. The strong inhibitory effect displayed by NAC against BACE1 increase and Aβ accumulation in human neuron-like cells provides a mechanistic rationale for new preventative and therapeutic strategies of sporadic AD. The therapeutic efficacy of NAC is, in fact, presently under investigation in a number of psychiatric disorders characterized by oxidative stress, including schizophrenia, nicotine and cocaine addiction, and obsessive-compulsive syndrome (Dean *et al*., 2011). Moreover, the treatment of patients with AD with certain nutraceutical formulations containing NAC exerts a significant cognitive improvement in comparison with placebo. A number of clinical trials are now in progress to confirm the protective action of NAC (Berk *et al*., 2013).

Our findings link an impaired cholesterol oxidative metabolism to an excessive β-amyloidogenesis and point to NAC as an efficient inhibitor of oxysterols-induced Aβ toxic peptide accumulation in the brain.

## Experimental procedures

### Cell culture, differentiation, and treatments

SK-N-BE neuroblastoma cells were maintained in Roswell Park Memorial Institute (RPMI) 1640 medium containing 2 mm glutamine and supplemented with 10% fetal bovine serum, 1% nonessential aminoacids, and 1% antibiotic mixture (penicillin–streptomycin–amphotericin) in a humidified atmosphere at 37 °C with 5% CO_2_. For differentiation, 2 × 10^6^ cells were plated in 75 cm^2^ culture flasks (Costar, Lowell, MA, USA) and exposed to 10 μm all-*trans*-retinoic acid for 10 days. The growth medium was changed thrice weekly.

Cells were treated with 1 μm 27-OH or with 1 μm 24-OH (Steraloids, Newport, RI, USA), both dissolved in ethanol. In some experiments, cells were pretreated with 100 μm NAC 1 h before the oxysterol treatments. Incubation times for all experiments are reported in the Results section and Figure legends.

In an additional set of experiments, the oxysterol concentration used in this study (1 μm) was compared to those present in the relevant literature (5 and 10 μm) with regard to their effect on Aβ_1–42_ production in both differentiated and undifferentiated SK-N-BE cells.

### Neuropathological characterization of AD and control brains

Brains were obtained from hospitalized patients at the Institute of Neurology Carlo Besta (Milan, Italy). In the brains included into the present study, routine neuropathological examination excluded relevant lesions such as tumors, significant vascular disease/stroke, inflammation, while revealed the presence of significant AD pathology, in terms of Aβ deposits and neurofibrillary changes.

The two AD hallmarks were searched by immunohistochemistry using antibodies against Aβ (4G8 1:4000; Signet Laboratories, Dedham, MA, USA) after formic acid pretreatment for 30 min and phospho-tau (AT8 1:300; Innogenetics, Ghent, Belgium). The immunoreaction was visualized using the EnVision Plus/Horseradish Peroxidase system (Dako Italia SpA, Milano, Italy) and 3-3′-diaminobenzidine as chromogen.

The brains were classified based on Braak and Braak staging system of neurofibrillary pathology (Braak & Braak, 1991). Six brains resulted at stage 1 or 2 (age at death from 72 to 86 years), and six brains were at stage 4–6 (age at death from 68 to 82 years). In the four brains used as controls (age at death from 25 to 71 years), the presence of Aβ and tau pathology was excluded.

### Oxysterol quantification in brain tissue

All autoptic samples were obtained between 24 and 36 h after death, and frontal cortex aliquots for oxysterols’ measurements were immediately washed with phosphate-buffered saline (PBS) to remove contaminating blood and stored at −80 °C. Oxysterols were measured by isotope dilution mass spectrometry essentially as previously described (Iuliano *et al*., 2003) with the exception that 25,26,26,26,27,27-hexadeuterocholest-5-ene-3ß,27-diol, and 25,26,26,26,27,27,27-heptadeuterocholest-5-ene-3ß,24-diol (Avanti PolarLipids, Alabaster, AL, USA) were used as internal standards, and the solid-phase extraction (SPE) step was repeated twice to eliminate cholesterol. The mass spectrometer was set to the selected ion monitoring mode; the ions used for analysis were as follows: [^2^H_6_]-27-hydroxycholesterol 463 *m*/*z*, [^2^H_6_]-24-hydroxycholesterol 463 *m*/*z*, 27-hydroxycholesterol 456 *m*/*z*, and 24-hydroxycholesterol 456 *m*/*z* (Avanti PolarLipids). Quantification of oxysterols was made by the internal standard ratio method.

### Preparation of cell lysates

Confluent differentiated cells were treated under the appropriate experimental conditions and placed immediately on ice-cold PBS. Whole-cell extracts were prepared in ice-cold lysing buffer [1 mL of PBS was fortified with 10 μL Triton X 100, 10 μL SDS 10%, 5 μL dithiotreitol (DTT) 1 m, 6 μL phenylmethylsulfonylfluoride 0.1%, and 10 μL aprotinin] for 20 min. The lysates were cleared by centrifugation at 14 000 *g* for 25 min. The protein concentration was measured following Bradford’s method (1976).

### RNA extraction and cDNA synthesis

Total RNA was extracted using TRIzol Reagent (Applied Biosystems, Monza, Italy) following the manufacturer’s instructions. RNA was dissolved in RNAse-free water fortified with RNAse inhibitors (RNase SUPERase-In; Ambion, Austin, TX, USA). The amount and purity (A260/A280 ratio) of the extracted RNA were assessed spectrophotometrically. cDNA was synthesized by reverse transcription from 2 μg RNA with a commercial kit and random primers (High-Capacity cDNA Reverse Transcription Kit; Applied Biosystems) following the manufacturer’s instructions.

### Real-time RT–PCR

Singleplex real-time RT–PCR was performed on 30 ng of cDNA using TaqMan Gene Expression Assay kits prepared for human APP, BACE1, PS1, ADAM10 and β2-microglobulin, TaqMan Fast Universal PCR Master Mix, and 7500 Fast Real-Time PCR System (Applied Biosystems). Negative controls did not include cDNA. The oligonucleotide sequences are not revealed by the manufacturer because of proprietary interests. The cycling parameters were as follows: 20 s at 95 °C for AmpErase UNG activation, 3 s at 95 °C for AmpliTaq Gold DNA polymerase activation, 40 cycles of 3 s at 95 °C (melting), and 30 s at 60 °C (annealing/extension). The fractional cycle number (Ct) at which fluorescence passes the threshold in the amplification plot of fluorescence signal versus cycle number was determined for each gene considered. The results were then normalized to the expression of β2-microglobulin, as housekeeping gene. Relative quantification of target gene expression was achieved with a mathematical method proposed by Livak and Schmittgen (2001).

### Antibodies and immunoblot analysis

The following antibodies were used: polyclonal antibody specific for 22 amino acids of the c-terminus of APP (Zymed Laboratories, Inc., San Francisco, CA, USA); polyclonal BACE1 antibody (Millipore, Temecula, CA, USA); polyclonal CTF-PS1 antibody (Cell Signaling Technology, Beverly, MA, USA); and polyclonal ADAM10 antibody (Santa Cruz Biotechnology Inc., Santa Cruz, CA, USA).

Total lysates were subjected to sodium dodecylsulfate-polyacrilamide gel electrophoresis on 9.3% acrylamide gels, using the mini-PROTEAN II electrophoresis cell (BioRad, Hercules, CA, USA). Proteins were transferred onto nitrocellulose membranes (Hybond-C extra; GE Healthcare, Arlington Heights, IL, USA). Nonspecific binding was blocked with 5% nonfat dry milk in 50 mm Tris-HCl, pH 7.4, containing 200 mm NaCl and 0.5 mm Tween-20 (Tris-buffered saline Tween). The blots were incubated with various different primary antibodies, followed by incubation with peroxidase-conjugated anti-mouse or anti-rabbit immunoglobulins in Tris-buffered saline Tween containing 2% nonfat dry milk. Reactions were developed with an enhanced chemiluminescence system following to the manufacturer’s protocol (GE Healthcare Biotech Italia, Cologno Monzese, Italy).

### Evaluation of Aβ_1–42_ production by ELISA

After cell treatment, whole-cell extracts were prepared in ice-cold lysing buffer (1 mL PBS was fortified with 10 mL TritonX-100, 10 mL SDS 10%, 5 mL DTT 1 m, 6 mL PMSF 0.1%, and 10 mL aprotinin) for 30 min and sonicated for 1 min. The lysates were then cleared by centrifugation at 17 860 *g* for 15 min. The protein concentration was measured following Bradford’s method (1976). Aβ_1-42_ levels were quantified using the Human/Rat βAmyloid (42) ELISA Kit (Wako Chemicals GmbH, Neuss, Germany) following the manufacturer’s instructions.

### Determination of β-secretase (BACE1) activity

The activity of BACE1 was determined using a commercially available secretase kit (Calbiochem, Merck, Darmstadt, Germany), following the manufacturer’s protocol. Cells were lysed in cold 1× Extraction buffer (ready for use in the kit) to yield a final protein concentration of mg mL^−1^. The method is based on the secretase-dependent cleavage of a secretase-specific peptide conjugated to the fluorescent reporter molecules EDANS and DABCYL, which results in the release of a fluorescent signal that can be detected on a fluorescence microplate reader (excitation wavelength 355 nm, and emission wavelength 510 nm). The secretase enzymatic activity is proportional to the fluorimetric reaction. Data were expressed as percentage change versus activity of control cells.

### Determination of γ-secretase activity

Cells were lysed in a hypotonic buffer containing 10 mm Tris–HCl, pH 7.4, 1 mm EGTA, and 1 mm EDTA. To extract the dissolved proteins, samples were centrifuged at 12 000 *g* for 20 min, and the supernatants were collected. To measure the enzymatic activity, 20 μg proteins were incubated with 20 μm of a fluorescent conjugated peptide substrate (NMA-GGVVIATVK (DPN)-DRDRDR-NH2) (Calbiochem, Merck) at 37 °C for 2 h. The degree of substrate cleavage was measured by the emitted fluorescence, using a reader Perkin-Elmer LS-55 (Perkin-Elmer, Waltham, MA, USA) with an excitation wavelength of 355 nm and an emission wavelength of 440 nm. Data were expressed as percentage change versus activity of control cells.

### Statistical analysis

All values are expressed as means ± standard deviation (SD). Data were assessed using one-way ANOVA with Bonferroni’s post-test for multiple comparisons. Differences at *P* < 0.05 were considered statistically significant. Calculations were made with graphpad instat3 software (GraphPad Software Inc., San Diego, CA, USA).

## Supporting Information

**Fig. S1** Intracellular Aβ_1-42_ accumulation modulated by 27-hydroxycholesterol (27-OH) and 24-hydroxycholesterol (24-OH) in differentiated or undifferentiated SK-N-BE cells.
