# Supplementary figures and images for: Up-regulation of β-amyloidogenesis in neuron-like human cells by both 24- and 27-hydroxycholesterol: protective effect of N-acetyl-cysteine
Source: Aging Cell. 2014 Feb 25;13(3):561–72. doi: 10.1111/acel.12206 (PMC4326893; doi:10.1111/acel.12206)

**A****Differentiated SK-N-BE**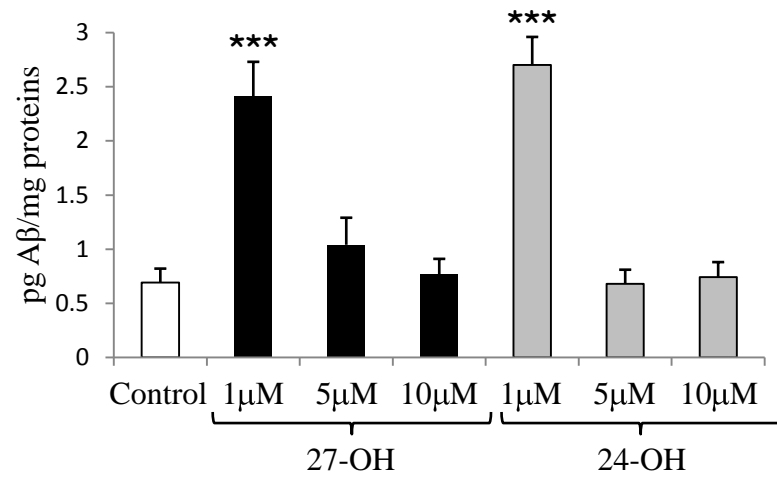**B****Undifferentiated SK-N-BE**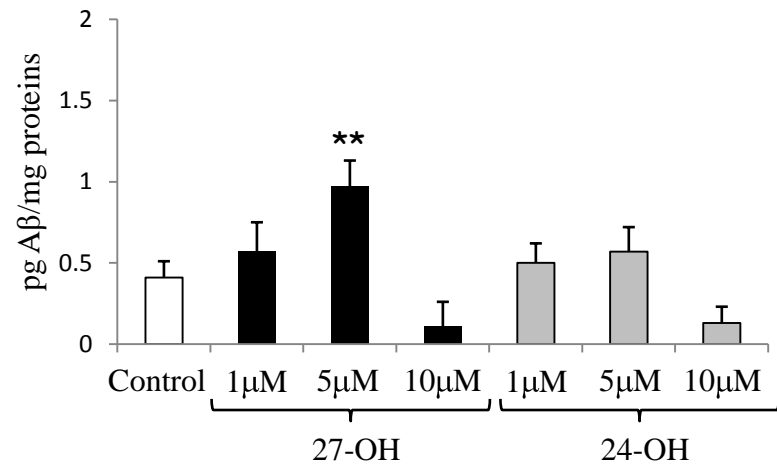

Supplement: Supplementary file 1 — Fig. S1 Intracellular Aβ1-42 accumulation modulated by 27-hydroxycholesterol (27-OH) and 24-hydroxycholesterol (24-OH) in differentiated or undifferentiated SK-N-BE cells. [file acel0013-0561-sd1.pdf]
