# Supplementary material for: Up-regulation of β-amyloidogenesis in neuron-like human cells by both 24- and 27-hydroxycholesterol: protective effect of N-acetyl-cysteine
Source: Aging Cell. 2014 Feb 25;13(3):561–72. doi: 10.1111/acel.12206 (PMC4326893; doi:10.1111/acel.12206)
Supplement: Supplementary file 2 [file acel0013-0561-sd2.docx]

**Fig. S1** Intracellular Aβ_1-42_ accumulation modulated by 27-hydroxycholesterol (27-OH) and 24-hydroxycholesterol (24-OH) in differentiated or undifferentiated SK-N-BE cells. SK-N-BE cells, differentiated or not, were incubated for 24 h with different concentrations of 27-OH or 24-OH (1, 5 or 10 μM). Untreated cells were used as control. Aβ_1-42_ concentration was quantified by enzyme-linked immunoassay (ELISA). Histograms represent the mean values ± SD of three experiments. ***P<0.001 and **P<0.01 vs. control group.
